# Complete genome of *Nakamurella* sp. PAMC28650: genomic insights into its environmental adaptation and biotechnological potential

**DOI:** 10.1007/s10142-022-00937-6

**Published:** 2022-12-23

**Authors:** Lakshan Paudel, Nisha Ghimire, So-Ra Han, Hyun Park, Sang-Hee Jung, Tae-Jin Oh

**Affiliations:** 1 Department of Life Science and Biochemical Engineering, Graduate School, Sun-Moon University, Asan, 31460 Korea; 2 Division of Biotechnology, College of Life Science and Biotechnology, Korea University, Seoul, 02841 Korea; 3 Department of Dental Hygiene, Gangneung Yeongdong University, Gangneung, 25521 Korea; 4 Genome-Based Bio-IT Convergence Institute, Asan, 31460 Korea; 5 Department of Pharmaceutical Engineering and Biotechnology, Sun-Moon University, Asan, 31460 Korea

**Keywords:** Complete genome, Glacier, CAZyme, Mycosporines, Isorenieratene, Cold adaptation, UV radiation

## Abstract

The mechanisms underlying the survival of bacteria in low temperature and high radiation are not yet fully understood. *Nakamurella* sp. PAMC28650 was isolated from a glacier of Rwenzori Mountain, Uganda, which species belonged to *Nakamurella* genus based on 16S rRNA phylogeny, ANI (average nucleotide identity), and BLAST Ring Image Generator (BRIG) analysis among *Frankineae* suborder. We conducted the whole genome sequencing and comparative genomics of *Nakamurella* sp. PAMC28650, to understand the genomic features pertaining to survival in cold environment, along with high UV (ultraviolet) radiation. This study highlights the role of polysaccharide in cold adaptation, mining of the UV protection-related secondary metabolites and other related to cold adaptation mechanism through different bioinformatics tools, and providing a brief overview of the genes present in DNA repair systems. *Nakamurella* sp. PAMC28650 contained glycogen and cellulose metabolism pathways, mycosporine-like amino acids and isorenieratene-synthesizing gene cluster, and a number of DNA repair systems. Also, the genome analysis showed osmoregulation-related genes and cold shock proteins. We infer these genomic features are linked to bacterial survival in cold and UV radiation.

**Supplementary Information:**

The online version contains supplementary material available at 10.1007/s10142-022-00937-6.

## Background

*Nakamurella*, a genus of the *Nakamurellaceae* family, was first highlighted by Yoshimi et al., with the illegitimate genus name “*Microsphaera*” (Yoshimi et al. 1996). *Nakamurellaceae* belongs to the suborder *Frankineae*, which consists of Gram-positive bacteria that help in nitrogen fixation. They are symbiotic prokaryotes with mild resistance to heavy metals (El dein Abdel-lateif et al. 2018). Japanese microbiologist Kazonuri Nakamura changed the illegitimate genus name to the logical and validly published name *Nakamurella* in 2004 (Tao et al. 2004). The characteristic features of *Nakamurella* are Gram-positive, aerobic, non-motile, non-spore forming, spherical, or irregular-short-rod-shaped actinobacteria. *Nakamurella* genus contains a validly published twelve species till now. They are *Nakamurella lactea* DLS-10, *Nakamurella aerolata* DB0629, *Nakamurella albus* YIM 132,087, *Nakamurella antarctica* S14-144, *Nakamurella deserti* 12Sc4-1, *Nakamurella flava* N5BH11, *Nakamurella intestinalis* 63 MJ, *Nakamurella panacisegetis* P4-7, *Nakamurella endophytica*, *Nakamurella silvestris* S20-107, *Nakamurella multipartita*, and *Nakamurella* sp. PAMC28650 (all information taken from the NCBI (National Centre for Biotechnology Information) database).

*Nakamurella* species have been isolated from different sources (Table 1), and their genomes are held responsible for their survival in different environmental conditions. The type strain of *Nakamurella* species, *Nakamurella multipartita*, was isolated from activated sludge acclimated with sugar-containing synthetic wastewater and characterized as a polysaccharide-accumulating Gram-positive coccus-shaped strain, whereas *Nakamurella antarctica* s14-144 was isolated from soil near the Antarctic Peninsula. *Nakamurella* sp. PAMC28650 was isolated from the glaciers of Rwenzori Mountain in Uganda. The Rwenzori Mountains are situated on the border between the Democratic Republic of Congo and Uganda, and form one of the largest glaciated regions, and the most extensive alpine glacier environment in Africa. Since our strain was isolated from glacier, and due to the extreme environment of the isolated sites, this candidate might be suitable in the field of the biodegradation and production of various industrially relevant compounds (Dumorné et al. 2017).

**Table 1:** List of draft genome of *Nakamurella* species along with their source of isolation

Species	Strain	Accession (NCBI)	Isolation source	Reference
*Nakamurella lactea*	DSM-19367	NZ_AUFT00000000	Rock	Kim et al. 2012
*Nakamurella aerolata*	DB0629	NZ_WLYK00000000.1	Air conditioner	Chaudhary et al. 2021
*Nakamurella albus*	YIM 132,087	NZ_WLYK00000000.1	Lichen	Jiang et al. 2020
*Nakamurella deserti*	12Sc4-1	NZ_QCXS00000000.1	Taklamakan desert	Liu et al. 2019
*Nakamurella flava*	N5BH11	NZ_SZZH00000000	Plant	Yan et al. 2020
*Nakamurella intestinalis*	63 MJ	NA	Feces of *Pseudorhynchus japonicus*	Kim et al. 2017
*Nakamurella endophytica*	2Q3S-4–2	NA	Plant tissue	Tuo et al. 2016
*Nakamurella silvestris*	S20-107	NA	Alpine forest soil	França et al. 2016
*Nakamurella panacisegetis*	P4-7	NZ_LT629710	Soil	Kim et al. 2012
*Nakamurella flavida*	DSM 26,917	NZ_JAERWL000000000.1	NA	NA
*Nakamurella antarctica*	S14-144	NZ_CP034170.1	Soil	Da et al. 2019
*Nakamurella* sp.	HJP_bin23	JAFIHI000000000.1	Sludge from phenol-fed wastewater aerobic bioreactor	NA
	Chersky.9_22	JADGOY000000000.1	Permafrost soil	NA

All the information was taken from NCBI database

*NA*, not available

Glacier regions with extremely harsh environment and subzero temperature are home to several microbiomes. They are a major source for the cold-adapted microorganisms (Anesio et al. 2017). These organisms cope with harsh environmental conditions, like freeze–thaw cycle, nutrient limitations, high amount of salt (salinity), and low temperature (Sakamoto and Murata 2002; Anesio et al. 2017). Besides that, cold-adapted bacteria show structural adjustment of enzymes, cold shock protein expression, membrane fluidity maintenance, presence of compatible solutes, and translation and transcription machinery adaptation to cope with external environment (Barria et al. 2013). Due to these factors, the isolation of the psychrophilic microorganisms may provide information about their unique genomic features, ability to produce different cold-adapted enzymes, and their genetic adaptability. At low temperature, the solubility of oxygen is higher, producing the higher reactive oxygen species (ROS), which provides oxidative stress. Because of this reason, oxidative metabolic pathways such as glycolysis, pentose phosphate pathway, tricarboxylic acid cycle (TCA), and electron transport chain are diminished at cold temperature. However, bacterial adjustment pathways with production of ROS are known, and the underlying molecular mechanism and alternative metabolic pathways in cold environment are not fully covered till now (Piette et al. 2012). CAZymes, on the other hand, might also play a significant role in the survival of the microorganisms in freezing temperature. Polysaccharides, such as glycogen, trehalose, and maltodextrin, are major carbohydrates present in cold-adapted microorganisms, and bacteria with their complete metabolic pathways indicate the ability to conserve energy and its utilization (Han et al. 2021). Psychrophilic microorganisms are also affected by high UV radiation, oxidative stress, and desiccation (Lauritano and Rizzo 2020). Pigments produced by cold-adapted microbes are considered as an energy source for the process of combating tools against the oxidative stress, extreme temperature, and safety tool against UV irradiation (Becker-Hapak et al. 1997). Depending upon the concentration and composition, the pigments are visible because of the color such as green (chlorophylls), yellow (xanthophylls), and orange to red (carotenoids) (Anesio et al. 2017) activities. Isorenieratene is a light-harvesting pigment carotenoid and generally synthesized by photosynthetic, green sulfur bacteria (Damsté et al. 2001). Naturally synthesizing microorganisms from cold areas have triggered great interest because of their importance in various industries such as nutraceutical, dairy, textile, cosmetics, and pharmaceutical. Furthermore, such pigments have different functions and may exhibit biotechnological activities differently such as photoprotective function, sunscreen, and antibacterial (Silva et al. 2021). Therefore, genomic study of this microorganism is likely to reveal the mechanisms of their adaptation to such harsh conditions.

Recently, with the ease in genome sequencing, complete genome analysis research is plentiful. However, the comparative genomic study of *Nakamurella* and their environmental adaptation features has not yet been reported. This paper presents the genomic analysis of *Nakamurella* sp. PAMC28650 isolated from glacier, to determine their environmental adaptation features with regard to carbohydrate metabolism, secondary metabolite production, and DNA repair systems.

## Materials and methods

### Isolation of *Nakamurella* sp. PAMC28650 and genomic DNA preparation

We received *Nakamurella* sp. PAMC28650, isolated from the glacier in Mt. Rwenzori, Uganda, from the Korea Polar Research Institute (KOPRI, Incheon, Korea). The 16 s rRNA of *Nakamurella* sp. PAMC28650 was subjected to EzBioCloud database (Yoon et al. 2017) and confirmed as the *Nakamurella* species. For complete genome analysis, the genomic DNA of *Nakamurella* sp. PAMC28650 was extracted using a QIAamp DNA Mini Kit (Qiagen Inc., Valencia, CA, USA). The quantity and purity were determined by spectrophotometry (Biochrome, Libra S35PC, UK). Also, we processed the genome to decide the quality by using A260/A280. The extracted genomic DNA was checked by agarose gel electrophoresis to evaluate its quality and stored at − 20 °C. The strain grew enough cells at 15 °C and then provided genomic DNA.

### Genome sequencing and assembly of *Nakamurella* sp. PAMC28650

Complete genome sequencing of *Nakamurella* sp. PAMC28650 was obtained by PacBio sequel single-molecule real-time (SMRT) sequencing technology (Pacific Biosciences, Menlo Park, CA, USA). SMRTbell library inserts (20 kb) were sequenced using SMRT cells. Results were generated from raw sequence data of 5,629,850 bp that were assembled de novo by using the hierarchical genome assembly process (HGAP) protocol (Chin et al. 2013) and HGAP4 assembly using SMRT analysis software (ver. 2.3; Pacific Biosciences, https://github.com/PacificBiosciences/SMRT-Analysis). The complete genome sequence was deposited in the GenBank database under the GenBank accession number CP060298.1.

### Genome annotation of *Nakamurella* sp. PAMC28650

Genome annotation of *Nakamurella* species was performed using the Rapid Annotation Subsystem Technology (RAST) server (Overbeek et al. 2014). All the genomes were downloaded using the National Center for Biotechnology Information (NCBI) in fasta format and uploaded to the server. Further, CAZyme analysis was conducted with the help of dbCAN tool (Zhang et al. 2018), and the Kyoto Encyclopedia of Genes and Genomes (KEGG) database (Kanehisa and Goto 2000), used to predict the pathways of cellulose and starch and glycogen metabolism. All the gene-encoding proteins were performed nucleotide BLAST analysis to know the conserved domain in conserved domain database (CDD). Circular map of the genomic DNA was made using the CGView comparison tool (Grant and Stothard 2008). For secondary metabolite gene clusters, genome fasta sequence was submitted to antiSMASH and RAST server, and the result was analyzed.

### Phylogenetic placement and comparative genome analysis

For phylogenetic analysis, we compared the genus belonging to *Frankineae* suborder including *Nakamurella* genus, using 16S rRNA. All the 16S rRNA sequences were downloaded from the NCBI database. MEGA X was used for the reconstruction of a neighbor-joining tree with 1000 bootstrap replicates (Kumar et al. 2018). We downloaded the complete genome sequences of *Frankia* suborder species available in GenBank (https://www.ncbi.nlm.nih.gov). First, the relationship of PAMC28650 with other *Frankia* suborder species was determined by comparing the value of average nucleotide identity (ANI), calculated using an OrthoANI in the Orthologous Average Nucleotide Identity Tool (OAT) (Lee et al. 2016). Three closely related *Nakamurella* spp. were selected for CAZyme analysis, and genes were reannotated by the DBCAN2 server. In addition, genome alignment of PAMC28650 was performed using BLAST Ring Image Generator (BRIG) (Alikhan et al. 2011).

## Results and discussion

### Complete genome profile of *Nakamurella* sp. PAMC28650

**Fig. 1:**
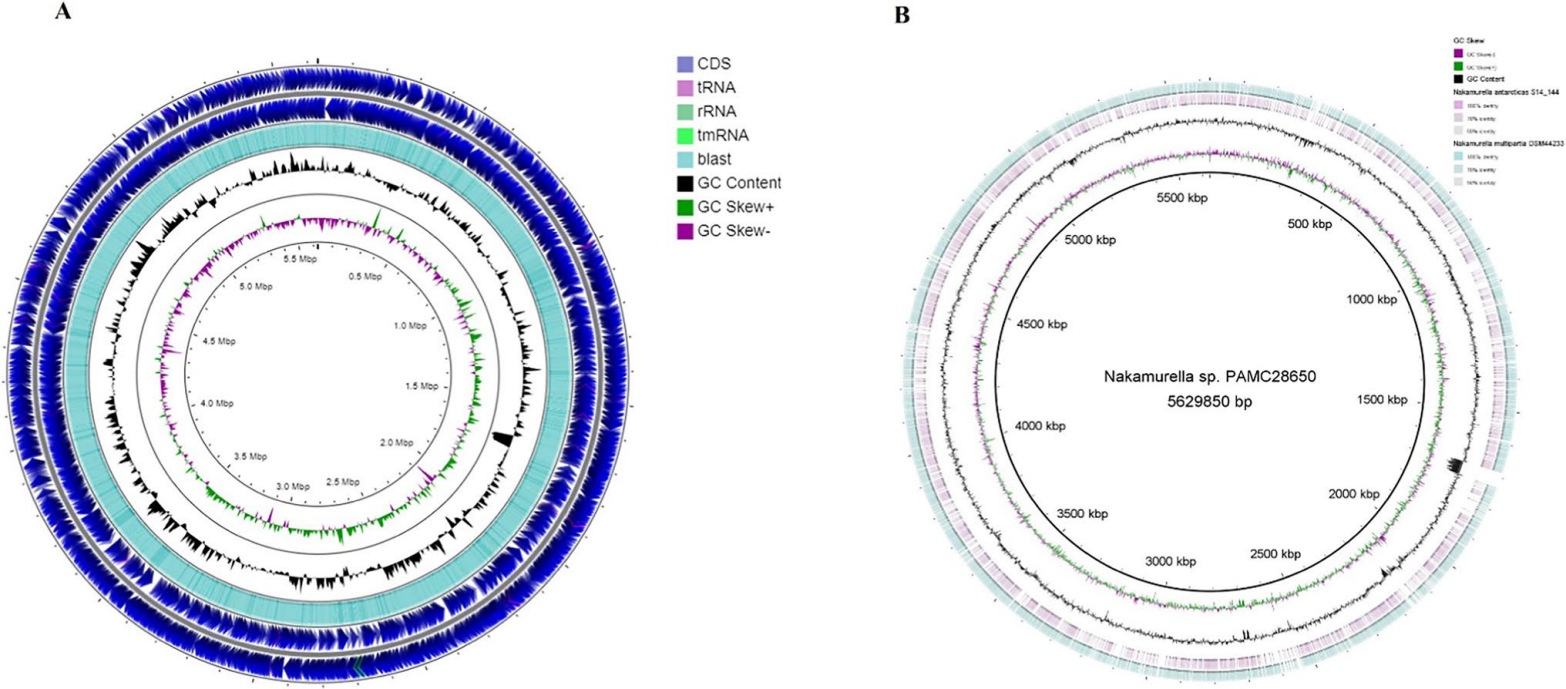
Genomic map and BRIG analysis. **A** Circular map and total genome information of *N.* sp. PAMC28650. Outer circle to inner circle: CDS, blast, GC content, GC skew + , GC skew − , and GC. **B** Comparison of complete nucleotide identity through blast analysis. Innermost layer is a *Nakamurella* sp. PAMC28650 (as a reference sequence) followed by *Nakamurella antarctica* s14-144 and *Nakamurella multipartita* DSM 44,233

*Nakamurella* sp. PAMC28650 has a circular chromosome of 5.63 Mb, as shown in Fig. 1A. It contains high GC percentage (67.9%). In this strain, the predicted protein-coding genes are total of 4888 including hypothetical protein. The predicted total numbers of tRNA and rRNA are 47 and 6, respectively, with Scaffold 1 (Table 2).

**Table 2:** General genome attributes of *Nakamurella* sp. PAMC28650

Category	*Nakamurella* sp. PAMC28650
Genome size (Mb)	5.63
Median GC%	67.9
rRNA	6
tRNA	47
Other RNA	3
Scaffold	1
Protein count	4888

**Fig. 2:**
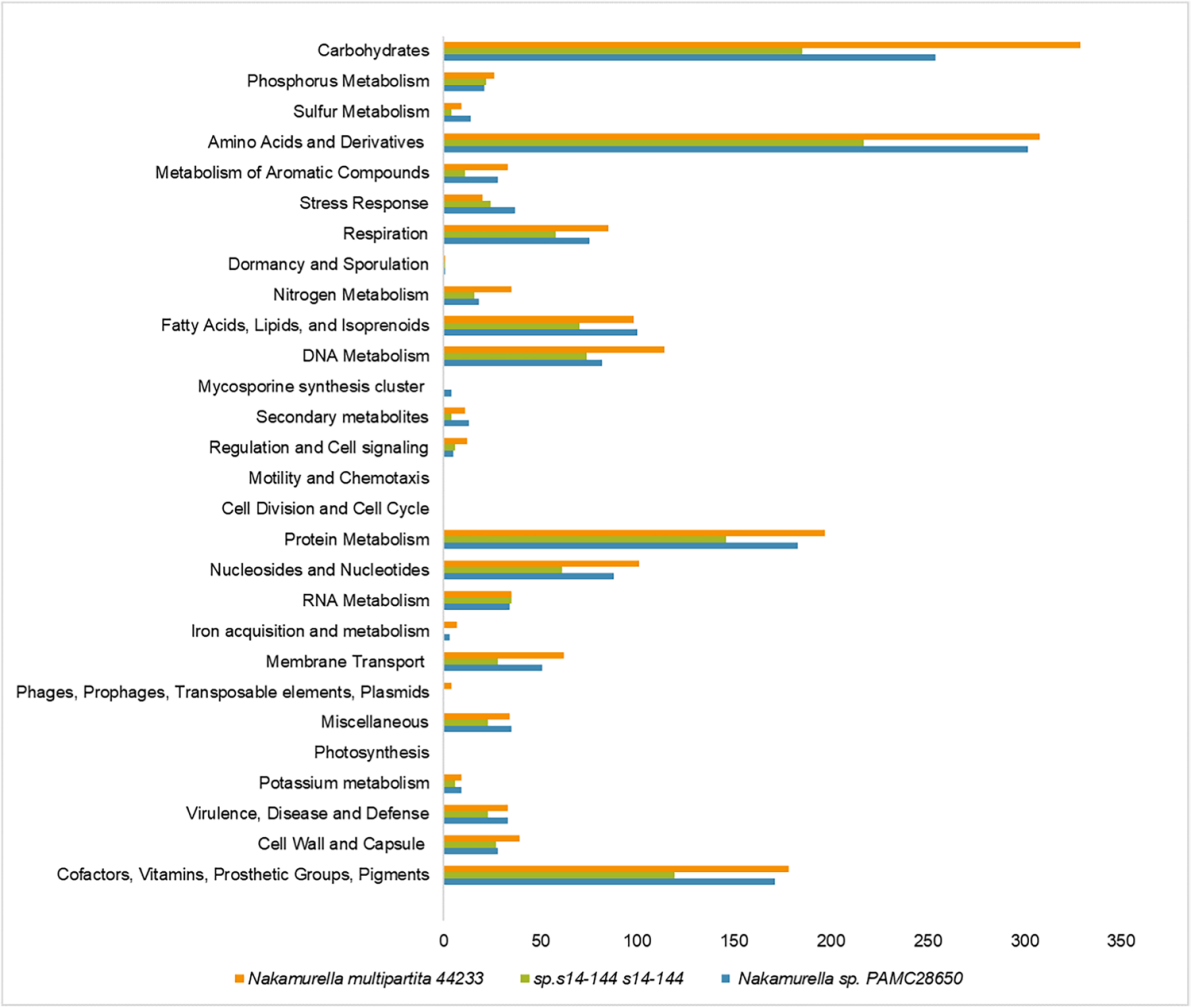
Comparison of the number of genes in different categories based on RAST annotation within the *Nakamurella* species. *X*-axis refers to the number of genes present in each category while *Y*-axis represents genes classified into different categories

The complete genome sequence of *Nakamurella* sp. PAMC28650 was subjected to RAST annotation server. The RAST subsystem is a manually curated SEED database and is therefore one of the most important bioinformatics tools used (Overbeek et al. 2014). RAST annotation categorizes the genes into two groups: either in the subsystem or not in the subsystem, depending on the protein families with common function. Genes that are categorized in the subsystem are considered reliable and conservative gene predictions. Among 5356 genes, only 20% (290) genes were classified in the subsystem (known functions), while 80% (4323) of genes were not in the subsystem (Table 3). Figure 2 compares the genes belonging to the subsystem between *Nakamurella* sp. PAMC28650, *Nakamurella multipartita* DSM44233, and *Nakamurella antarctica* s14-144. All *Nakamurella* species showed the highest number of genes in the categories like carbohydrates, amino acids and derivatives, protein metabolism and cofactors, vitamins, prosthetic groups, and pigments. Bacterial stress response (e.g., heat shock and cold shock response) was also an important factor to withstand the extreme surrounding, and therefore, PAMC28650 has the highest proportion of genes as compared to other *Nakamurella* species.

**Table 3:** Comparison of genomic features of fully sequence *Nakamurella* species

Species/category	*Nakamurella* sp. PAMC28650	*Nakamurella multipartita* DSM44233	*Nakamurella antarctica* s14-144
Group	This study	US DOE Joint Genome Institute (JGI-PGF)	Wuhan University
Genome size	5.63	6.06	3.38
Chromosome	1	1	1
Plasmid	0	1	0
G + C %	67.9	70.9	61.6
Protein-coding genes	4851	5227	2904
Accession number	CP060298.1	CP001737.1	CP034170.1
Isolation source	Glacier	Activated sludge cultured in fed-batch reactors	Soil
RAST analysis			
In subsystem	20%	21%	23%
Not in subsystem	80%	79%	77%

All the data were taken from the NCBI database. Subsystem information was taken from the RAST analysis

### Phylogenetic tree, ANI, and BRIG analysis of *Nakamurella* species

**Fig. 3:**
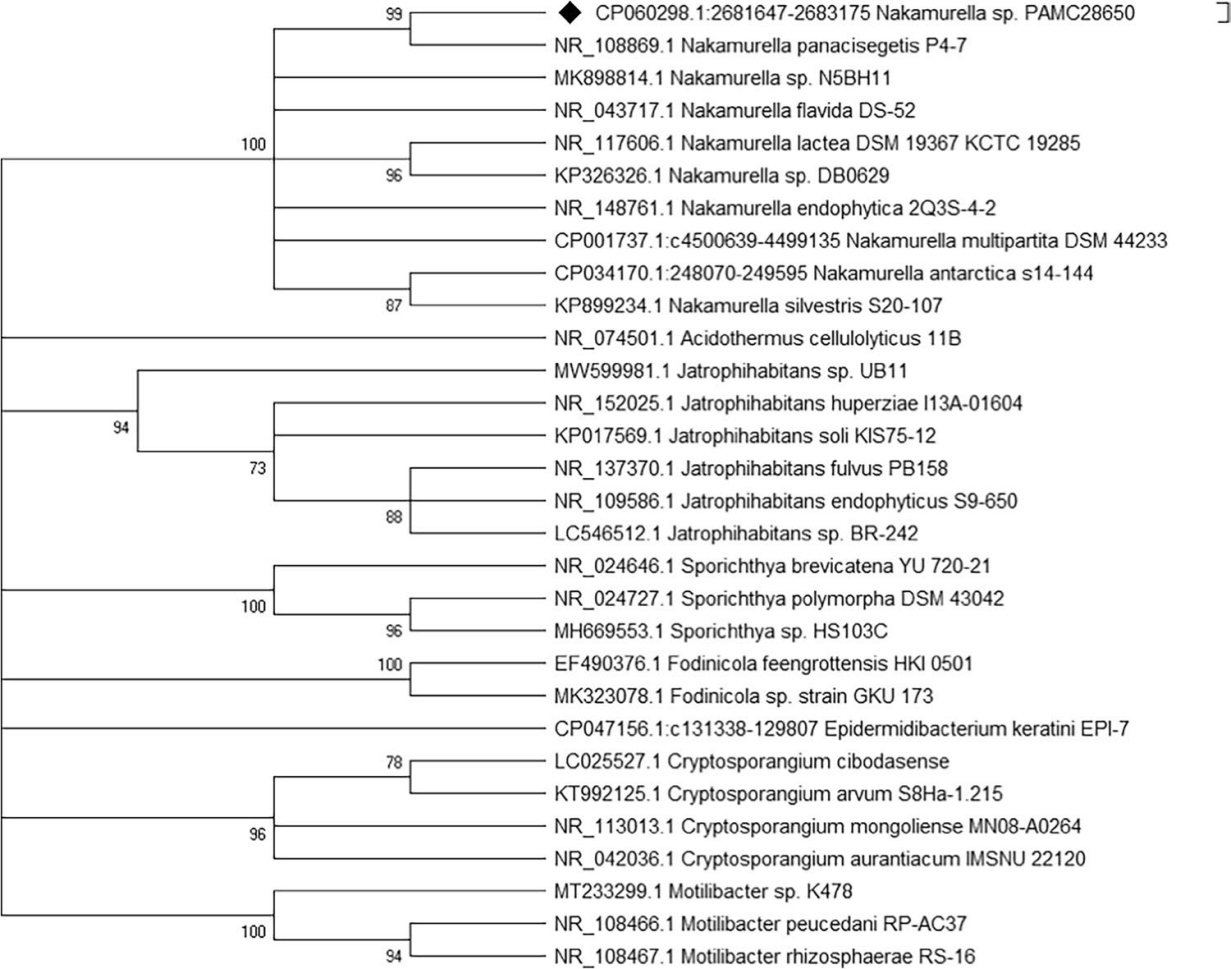
Bootstrap consensus tree developed by the maximum likelihood tree using 16S rRNA of the complete and draft genome of *Frankineae* suborder including *N.* sp. PAMC28650. The percentages of replicate trees (1000 replicates bootstrap test) among the clustered taxa are shown next to the branches. The colors represent different clades with common ancestors

The result obtained from EzBioCloud database for 16 s rRNA analysis is shown in Table S1, where the strain shows 99.3% pairwise similarity with *Nakamurella panacisegetis*. The phylogenetic position of *Nakamurella* sp. PAMC28650 was analyzed using 16S rRNA sequence of 31 species belonging to *Frankineae* suborder, including our strain. We included 16S rRNA of all the complete and partial genomes of *Nakamurella* species. All the *Nakamurella* species fell in the same clade with a common ancestor, while the closest relative of *Nakamurella* sp. PAMC28650 was found to be *Nakamurella panacisegetis* strain P4-7. We can say that this strain belongs to the family *Nakamurellaceae* and phylum *Actinobacteria*. Figure 3 shows the phylogenic position of our strain.

**Fig. 4:**
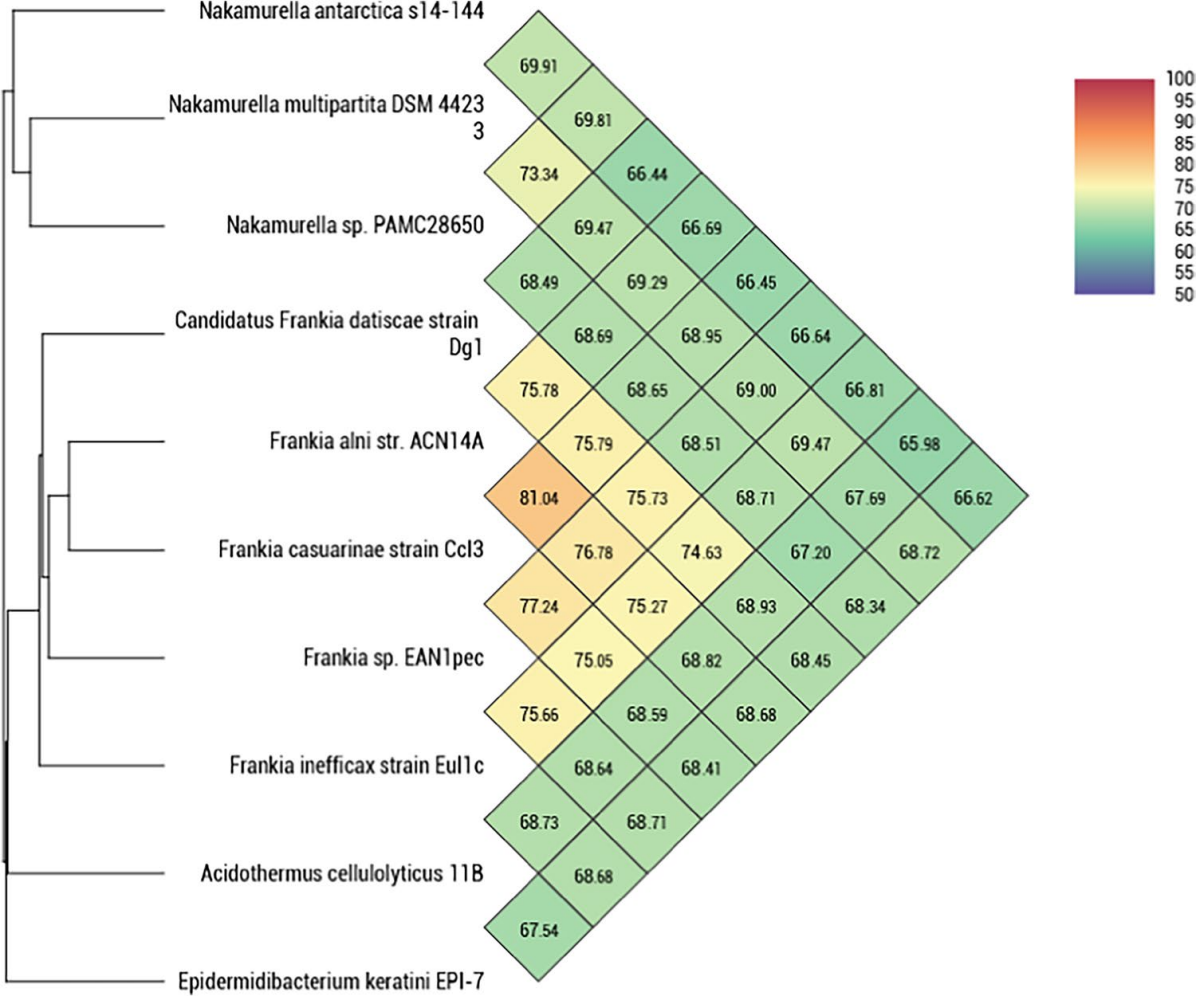
Average nucleotide identity analysis performed between the species having complete genomes within *Frankineae* suborder. Blue color indicates the lowest identity, whereas red color indicates the highest identity

Average nucleotide identity (ANI) is another way to determine the relatedness of *Nakamurella* sp. PAMC28650 strain, by comparison with the complete nucleotide sequences of the species from *Frankineae* suborder. ANI values were calculated using OrhtoANI in the Orthologous Average Nucleotide Identity Tool (OAT) with cutoff value (95 to 96%) (Lee et al. 2016). *Nakamurella* sp. PAMC28650 showed the highest ANI value with *Nakamurella multipartita* DSM44233 (73.34%) isolated from activated sludge acclimated with sugar-containing synthetic wastewater and *Nakamurella antarctica* s14*-*144 (69.81%) from soil (Fig. 4). Based on the ANI value, no significant identity was observed for *Nakamurella* sp. PAMC28650 among any species in the *Frankineae* suborder; therefore, it might belong to a new species (Table S4). We had only included *Nakamurella multipartita* DSM44233 and *Nakamurella antarctica* s14-144 among the *Nakamurella* species for ANI calculation, as complete nucleotide sequences of other *Nakamurella* species were not available in the NCBI. The ANI method is the common method for bacterial comparative genomics. This calculates the average base similarity between two different microbial genomes of homologous fragment at the nucleotide level like the DNA–DNA hybridization method, to identify the relatedness between bacterial species. We also compared the whole genome sequences of the *Nakamurella* sp. PAMC28650 as a reference strain including *Nakamurella multipartita* DSM44233 and *Nakamurella antarctica* s14-14 using BLAST Ring Image Generator (BRIG). BRIG analysis represents the circular genomic map showing the identity between genome of *Nakamurella* sp. PAMC28650 against *Nakamurella antarctica* s14-144 and *Nakamurella multipartita* DSM44233 in a circular ring, colored depending on the BLAST identity (Fig. 1B). This analysis also highlighted that the shared genome and unmatched genome sequences among three species indicating our strain may be a new species. BRIG analysis also supports the result of ANI analysis. Therefore, the *Nakamurella* sp. PAMC28650 may be a new species.

### Comparative CAZyme analysis and carbohydrate metabolism in cold-adapted *Nakamurella* species

**Fig. 5:**
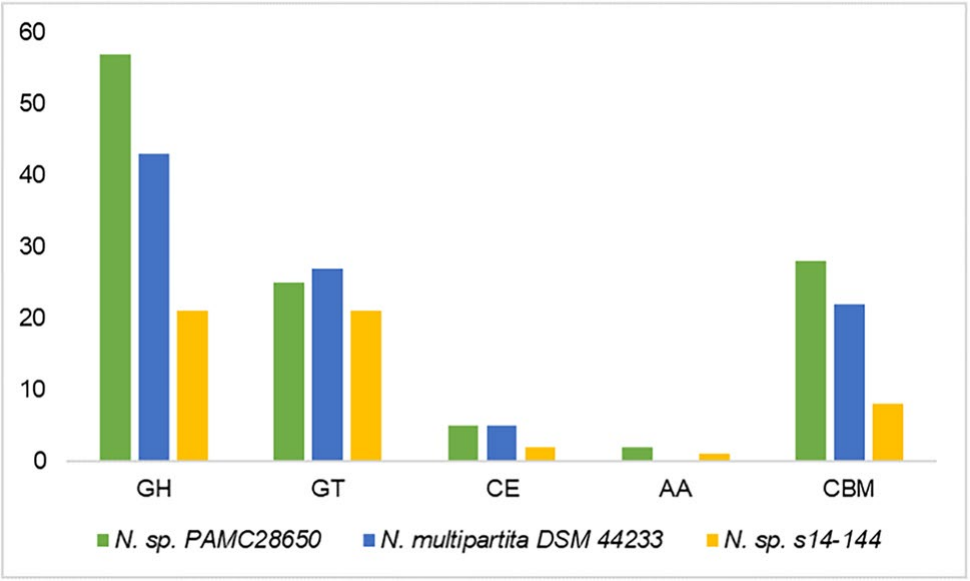
Distribution of CAZymes among the *Nakamurella* species. CAZymes are categorized into different groups based on the gene annotation. The colors represent differentiation among species. *X*-axis and *Y*-axis represent the different GH families and percentage of genes respectively. GH, glycosyl hydrolase; GT, glycosyltransferase; CE, carbohydrate esterase; PL, polysaccharide lyase; AA, axillary activities; CBM, carbohydrate-binding modules

**Fig. 6:**
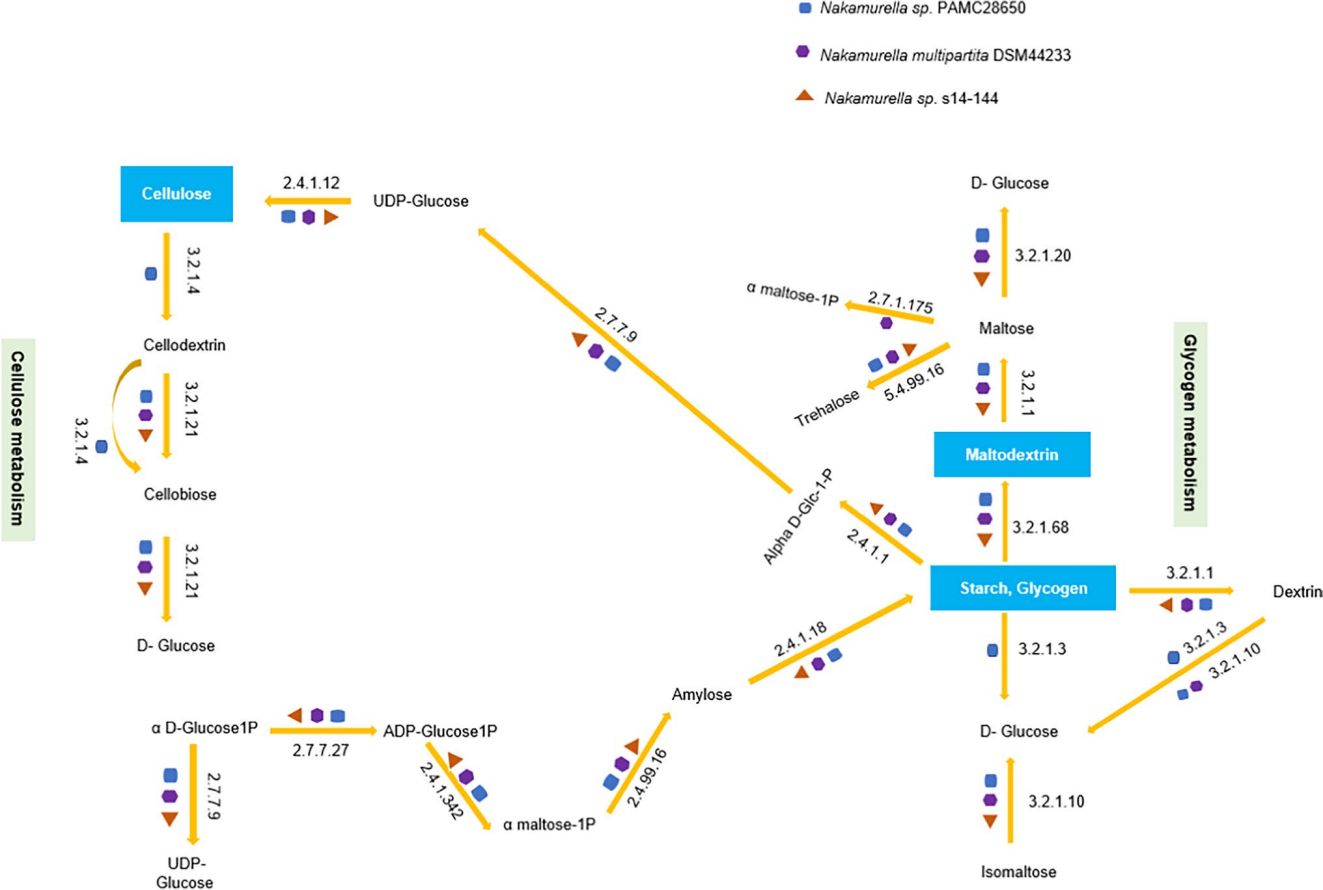
Comparison of predicted pathways for polysaccharide metabolism in *Nakamurella* species. The predicted intermediates of glycogen and cellulose metabolism pathway and respective enzyme’s EC number are taken from KEGG

CAZyme analysis was done to find out the carbohydrate metabolism-related genes in *Nakamurella* sp. PAMC28650 strain. The CAZyme-related genes predicted in this strain are categorized into 6 groups, namely, glycoside hydrolases (GH), glycosyltransferases (GT), polysaccharide lyases (PL), carbohydrate esterases (CE), carbohydrate-binding modules (CBM), and auxiliary activities (AA). Among the 117 CAZyme genes predicted by dbCAN database in PAMC28650 strain, the maximum numbers of genes annotated are in GH families (57), 28 genes in CBM family, 25 genes in GT family, and 2 in AA family (Tables S2 and S3). However, there are no genes classified in PL family. On comparison to *Nakamurella multipartita* DSM44233 and *Nakamurella antarctica* s14-144, *Nakamurella* sp. PAMC28650 contained maximum numbers of GH, CBM, and AA families (Fig. 5). Further analysis of the genome of *Nakamurella* sp. PAMC28650 strain for carbohydrate metabolism in KEGG database, genes, and pathways related to glycogen and cellulose metabolism was revealed. Figure 6 and Table 4 show the list of enzymes and their CAZyme classification for cellulose and glycogen metabolism.

**Table 4:** List of CAZymes in *Nakamurella* sp. PAMC28650 involved in glycogen and cellulose metabolism

dbCAN ID	Protein (CDD)	Gene	EC number	CAZyme family
NZ_CP060298.1_1474	Cellulase	*CLEB*	3.2.1.4	GH5
NZ_CP060298.1_1067	Beta glucosidase	*bg1X*	3.2.1.21	GH3
NZ_CP060298.1_2591	Cellulose synthase	*bcsA*	2.4.1.12	GH2
NZ_CP060298.1_74	UTP-glucose-1-phosphate uridylyltransferase	*glaF*	2.7.7.9	NA
NZ_CP060298.1_1297	Maltose alpha-D-glucosyltransferase/alpha-amylase	*glgE*	5.4.99.16	GH13-16
NZ_CP060298.1_1131	Alpha-amylase	*malS*	3.2.1.1	GH13
NZ_CP060298.1_3953	Maltase-glucoamylase	*MGAM*	3.2.1.20	GH15
NZ_CP060298.1_3360	Maltooligosyltrehalose trehalohydrolase	*glgZ*	3.2.1.141	GH13
NZ_CP060298.1_3174	(1- > 4)-Alpha-D-glucan 1-alpha-D-glucosylmutase	*glgY*	5.4.99.15	GH13-16
NZ_CP060298.1_4064	Isoamylase	*treX*	3.2.1.68	GH13
NZ_CP060298.1_	Maltase-glucoamylase	*MGAM*	3.2.1.3	GH31
NZ_CP060298.1_42	Sucrase-isomaltase	*SI*	3.2.1.10	GH31
NZ_CP060298.1_4414	Glycogen phosphorylase	*glgP*	2.4.1.1	GH32
NZ_CP060298.1_550	Maltokinase	*pep2*	2.7.1.175	NA
NZ_CP060298.1_3358	1,4-Alpha-glucan-branching enzyme	*glgB*	2.4.1.18	GH57
NZ_CP060298.1_3172	Alpha-1,4-glucan:maltose-1-phosphate maltosyltransferase	*glgE*	2.4.99.16	GH13-30
NZ_CP060298.1_309	Glucose-1-phosphate adenylyltransferase/ADP glucose pyrophosphorylase	*glgC*	2.7.7.27	NA
NZ_CP060298.1_2251	Alpha-maltose-1-phosphate synthase	*glgM*	2.4.1.342	GT4

*CDD*, conserved domain database; *EC*, enzyme commission number; *NA*, not available; *GH*, glycosyl hydrolase; *GT*, glycosyltransferase

Glycogen is a homopolysaccharide composed of alpha *D*-glucose held together by alpha-1,4 and alpha-1,6 glycosidic bond, which is mainly found in animals, fungi, and bacteria, and which acts as a major source of energy storage in bacteria (Wilson et al. 2010). Generally, the classical routes for glycogen synthesis in most of the bacteria include mainly three enzymes: adenosine diphosphate glucose pyrophosphorylase (AGPase; EC 2.7.7.27), glycogen synthase (GS; EC 2.4.1.21), and branching enzymes (GBE; 2.4.1.18) (Iglesias and Preiss 1992). However, all *Nakamurella* species under study lacked glycogen synthase; instead, they include alpha-maltose-1-phosphate synthase [EC 2.4.1.342] and alpha-1,4-glucan:maltose-1-phosphate [EC 2.4.99.16], resulting in the formation of amylose and 1,4-alpha-glucan-branching enzyme [EC 2.4.1.18] for finally synthesizing glycogen. *Arthrobacter* species PAMC25564 isolated from cold environment also has the similar genomic data with *Nakamurella* sp. for glycogen and maltodextrin synthesis pathway (Han et al. 2021). *Nakamurella* sp. PAMC28650 also contained glycogen utilization pathway. Intracellular glycogen utilization by bacteria has different routes. The classical pathway for glycogen degradation takes place by two key enzymes: glycogen phosphorylase [EC 2.4.1.1] and debranching enzymes (GDE; EC 3.1.1) (Cifuente et al. 2019). *Nakamurella* species have similar pathways with various debranching enzymes, as shown in the predicted glycogen degradation pathway (Table 4). Glycogen heterogeneities support the durable energy storage mechanism hypothesis (DESM) (Wang and Wise 2011), and glycogen with its slow degradation nature means the use of glucose by bacteria at a slow rate, so that bacteria can survive longer time in a starved condition.

Cellulose is a polysaccharide composed of a linear chain of beta *D*-glucose held together by beta-1,4 glycosidic bond (Updegraff 1969). For the synthesis of cellulose, UDP-glucose is polymerized with the help of enzyme cellulose synthase [EC 2.4.1.12] into long and unbranched chain (beta-1,4 chain) (Klemm et al. 2005). Naturally, bacteria can synthesize cellulose as a protective layer in the cells (Ross et al. 1991). Only *Nakamurella* sp. PAMC28650 contained cellulose synthase, but the cellulose degradation pathway was present in all the *Nakamurella* species studied (Fig. 6). Some of the bacterial species, including Gram-positive and Gram-negative bacteria, also produce cellulose (Jonas and Farah 1998). Both plants and bacteria can synthesize cellulose naturally, but their difference is that bacterial cellulose has high purity, and greater water holding capacity, surface area, degree of polymerization (Gorgieva and Trček 2019), and porosity (Al-Shamary and Darwash 2013), as compared to plant cellulose. Another aspect of cellulose biosynthesis in bacteria is that they are responsible for the mechanical and chemical, as well as physiological, protection (R. Rebelo et al. 2018). For the cellulose catabolism, cellulase is the key enzyme, and psychrophilic bacteria are the common bacteria to produce cellulase enzyme (Kasana and Gulati 2011). Cold-adapted microorganisms reside in a low-temperature environment, and in such environment, cellulose synthesis and its utilization considering the large proportion of carbon cycle (Garsoux et al. 2004; Zeng et al. 2006) discovered extracellular cold-active cellulase gene *celX* in deep-sea psychrotrophic *Pseudoalteromonas* sp. It typically consists of a catalytic domain (N-terminal; glycoside hydrolase family 5) and a cellulose-binding domain (C-terminal; CBM family 5), with cellobiose serving as the key hydrolysate. Similarly, the role of Cel5G, a psychrophilic cellulase from the Antarctic bacterium *Pseudoalteromonas haloplanktis*, was studied through protein engineering, where the unusually long flexible linker region was found to enhance catalytic efficiency in the cold region (Sonan et al. 2007). This supports that the cold-adapted cellulase helps bacteria retain metabolic and enzymatic activities in extreme environments by utilizing cellulose (Wang et al. 2019). Cellulase enzyme has diversified function, such as textiles and detergent additives; therefore, its isolation from cold-adapted bacteria and industrial production are quite demanding.

Overall, CAZyme analysis plays an important role in understanding the survival of cold-adapted organisms in extreme environments. Not much research has been conducted that particularly focuses on the survival of organisms in zero and subzero temperature in relation to carbohydrate metabolism. However, carbohydrate’s metabolisms, such as starch, glycogen, and trehalose metabolisms, are known as sources of energy for cold-tolerant microorganisms (Han et al. 2021). Another recently published paper also highlighted the importance of cellulose and hemicellulose metabolism for survival in harsh environment (Shen et al. 2021). During extreme environment, primary metabolism is diminished in bacteria, and very little energy is generated using carbohydrates. Both catabolism and anabolism are necessary to store and generate the energy for the survival of cold-residing microorganisms.

### Osmotic stress regulatory genes present in *Nakamurella* sp. PAMC28650

Bacteria reside different environmental conditions with continuous changing physical properties such as temperature, salinity, and osmolarity. Despite the variation in the external environment, microorganisms have to maintain cytoplasmic hydration for their growth and survival. Osmotic stress from the environment is one of the important factors that determine the ability of any microorganism to proliferate in its habitat. Osmotic stress implies an increase or decrease in the osmolarity of the environment of an organism, and organisms carry out the primary regulatory process to prevent it. To a high osmotic environment, the main response of bacteria is to accumulate various solutes such as potassium, glutamate, trehalose, proline, and glycine betaine at a concentration that is nearly equal to the osmolarity of the medium (Csonka 1989). The accumulation of compatible solutes can be achieved by either synthesis and/or transportation to maintain the structure of biological macromolecules. Multiple osmoregulatory transporters such as overlapping energy-coupling mechanisms, substrate specificity, and mechanosensitive channels are known to cope osmotic stress (Sukharev et al. 1997; Wood 1999; Booth and Louis 1999). Aquaporins are an osmotically inducible protein released in an osmoregulatory response, which is required for growing cells. Production of glycerol uptake facilitator proteins are also known to be in the response to osmotic pressure (Ben-Amotz and Avron 1973). Other important aspects of osmoregulation are betaine biosynthesis and choline-betaine uptake from the bacterial cell membranes. Glycine betaine biosynthesis is a two-step process (precursor is choline), and it is considered a very effective compatible solute against cold stress protectant. Alternatively, there are various types of glycine betaine transporters, such as OpuA, OpuC, and OpuD, in different bacteria, which helps to accumulate the solutes against osmotic stress (Hoffmann and Bremer 2011).

*Nakamurella* sp. PAMC28650 inherits five genes related to the osmoregulation, whereas *Nakamurella multipartita* DSM44233 and *Nakamurella antarctica* s14-144 contain one and two, respectively. Similarly, choline, betaine uptake, and betaine synthesis genes are also present in *Nakamurella* sp. PAMC28650. Generally, betaine biosynthesis requires two genes such as BetA (choline dehydrogenase) and BetB (betaine aldehyde dehydrogenase) for the synthesis of glycine betaine. BetA and BetB genes are present in both *Nakamurella* sp. PAMC28650 and *Nakamurella antarctica* s14-144 strains, but absent in *Nakamurella multipartita* DSM44233. Similarly, three different SOX related to sarcosine oxidase subunit genes are also present in both *Nakamurella* species except *Nakamurella antarctica* s14-144. Glycine betaine transporters OpuA three genes are present in our strains; however, *Nakamurella multipartita* DSM44233 contains one ProU gene. Overall, the number of genes is higher in *Nakamurella* sp. PAMC28650 compared to other *Nakamurella* strains (Table 5).

**Table 5:** Comparison of osmoregulation-related genes present in *Nakamurella* species

RAST ID	Function (osmoregulation)	CDD	*Nakamurella* sp. PAMC28650	*Nakamurella multipartita* DSM44233	*Nakamurella antarctica* s14-144
2,762,325.4, peg.2004, 2,762,325.4, peg.611	Aquaporins Z	cd00333	2 genes	1 gene	1 gene
2,762,325.4, peg.2024, 2,762,325.4, peg.2024, 2,762,325.4, peg.356	Glycerol uptake facilitator protein	cd00333, PTZ00016	3 genes	–	1 gene
Choline, betaine uptake, and betaine biosynthesis					
2,762,325.4, peg.1510	BetA	PRK02106	1 gene	–	1 gene
2,762,325.4, peg.1497	BetB	Cd07119	1 gene	–	1 gene
2,762,325.4, peg.1494, 2,762,325.4, peg.1495, 2,762,325.4, peg.1496	OpauA or ProU	Cd13643, Pfam00528, NF033858	3 genes	–	1 gene
2,762,325.4, peg.1489, 2,762,325.4, peg.1490, 2,762,325.4, peg.1491	SOX	TIGR01372, TIGR01372, TIGR01373	3 genes	–	3 genes

All the information were taken from the RAST server. Highlighted genes were compared among the *Nakamurella* species (– signs indicate the absence of gene and numbers indicate the available genes)

*peg.* protein-encoding genes, *CDD* conserved domain database

### Cold shock protein-related genes present in *Nakamurella* PAMC28650

A number of mechanisms are involved in microorganisms for adapting with various extreme environmental conditions for the microbial survival. Temperature is one of the key environmental factors affecting growth and the survival of microorganisms (Richter et al. 2010). Bacteria produce small cold shock proteins (multifunctional RNA-DNA-binding proteins) to counteract harmful effect of temperature downshift (Graumann and Marahiel 1996). Cold shock proteins (Csps) have been reported in psychrophilic, mesophilic, and even hyperthermophilic bacteria. Till now, only CspA, CspB, CspE, CspG, and Cspl proteins were induced from the *Escherichia coli* by cold shock (Jin et al. 2014).

Cell membrane homeostasis and activity of enzyme diminish in bacteria during cold shock, and this will ultimately reduce the efficacy of transcription and translation. Furthermore, the protein folding and function of ribosome are disturbed. Csps generally bind to both DNA and RNA to monitor the transcription process by promoting the formation of single-stranded RNA. It also enhances the translation of proteins through CspA, which helps to facilitate the selection of translation at low temperature (Keto-Timonen et al. 2016). In the *Nakamurella* sp. PAMC28650, three genes encoding two CspA and one CspE were categorized by the RAST analysis (Table S5). CspA is reported as the major shock protein whereas CspE has been reported for the regulation and expression of cold response protein. These genes share high similarity of more than 57% with the known Csps from the UniProt database. Like other different cold adaptational feature analyzed by genome analysis tool, these Csps might have role in adaptation in such harsh condition.

### Secondary metabolite gene cluster in *Nakamurella* sp. PAMC28650 and their relevance to UV resistance

**Fig. 7:**
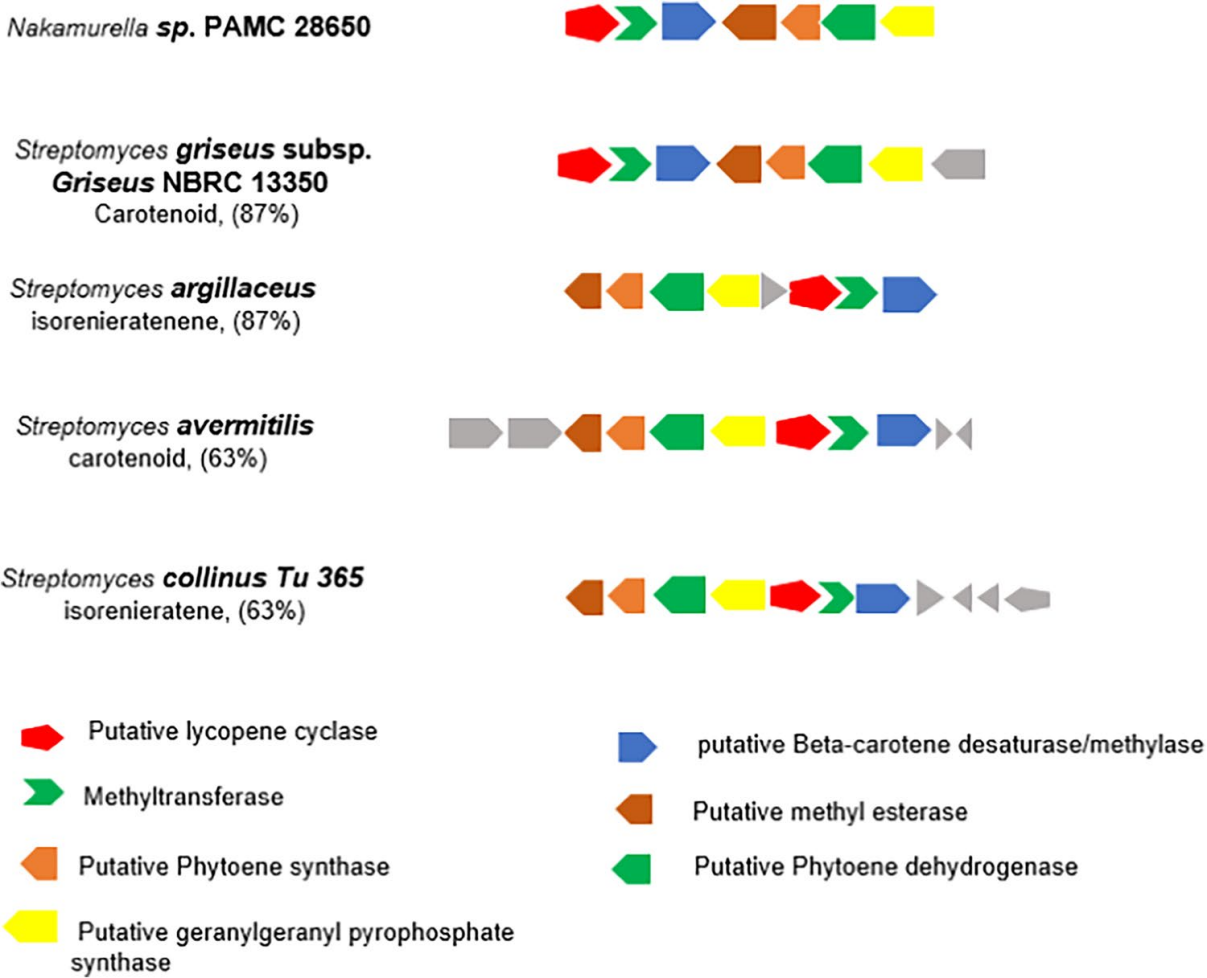
Putative isoreineratene biosynthetic gene cluster of *Nakamurella* sp. PAMC28650, compared with other already characterized bacterial species. Gray color denotes that the genes do not present in *Nakamurella* sp. PAMC28650 gene cluster

Secondary metabolites, which are not normally involved in cellular function such as growth, adhesion, development, and reproduction, are the organic compounds synthesized by plants, fungi, and bacteria. Bacteria produce different kinds of secondary metabolites during their stationary phase, because of nutritional deficient to overcome the environmental stress (Ruiz et al. 2010). *Nakamurella* sp. PAMC28650 was isolated from Mt. Rwenzori, Uganda, where evidence suggests a very high UV radiation impact on lakes in the Rwenzori Mountains. We analyzed the genome of *Nakamurella* sp. PAMC28650 along with other *Nakamurella* species to find genes related to secondary metabolites using RAST annotation server (Table S5) and antiSMASH. The secondary metabolite gene clusters distributed among all the species are shown in Table S6 and Fig. S1. PAMC28650 was found to contain gene cluster for the synthesis of isorenieratene and mycosporine-like amino acid (MAA). Both the metabolites are responsible for the protection of microorganisms from environment subjected to high UV radiation (Bhatia et al. 2011; Chen et al. 2019). Carotenoids are an important group of isoprenoid units, having a characteristic of conjugated tetraterpenes (C40). Its color ranges from red, yellow, orange, and colorless. Light-absorbing chromophores present in carotenoids play a crucial role in preventing UV radiation to the cells as well as showing antioxidant effects (Wang et al. 2019). Briefly, a carotenoid molecule may exchange electronic energy during energy quenching and change into triplet oxygen. Under these changes, it can easily prevent the oxidation reaction (Frank and Brudvig 2004). Isorenieratene is a light-absorbing carotenoid pigment produced by bacteria, fungi, and algae including plants (Yamaguchi 1959). It is an aromatic compound like beta-carotene, having two Φ rings that contribute to its better stability against oxidative stress. Benzene ring has been especially highlighted for UV resistance feature (Martin et al. 2009; Chen et al. 2018). *Nakamurella* sp. PAMC28650 possessed gene cluster for isorenieratene biosynthesis, where 87% of its genes show similarity with the already characterized isorenieratene gene cluster of *Streptomyces argillaceus* and similar percentage of gene similarity with carotenoid gene cluster of *Streptomyces griseus* subsp. *griseus* NBRC13350, as shown in Fig. 7. Figure 7 details all of the related enzymes and their gene cluster. Extremophilic bacteria, such as *Deinococcus-Thermus* group, *Bacillus* genus, and mesophilic bacteria, namely, *Escherichia coli*, have been studied to understand the role of carotenoids in UV resistance (Yoon et al. 2009; Tian and Hua 2010).

**Fig. 8:**
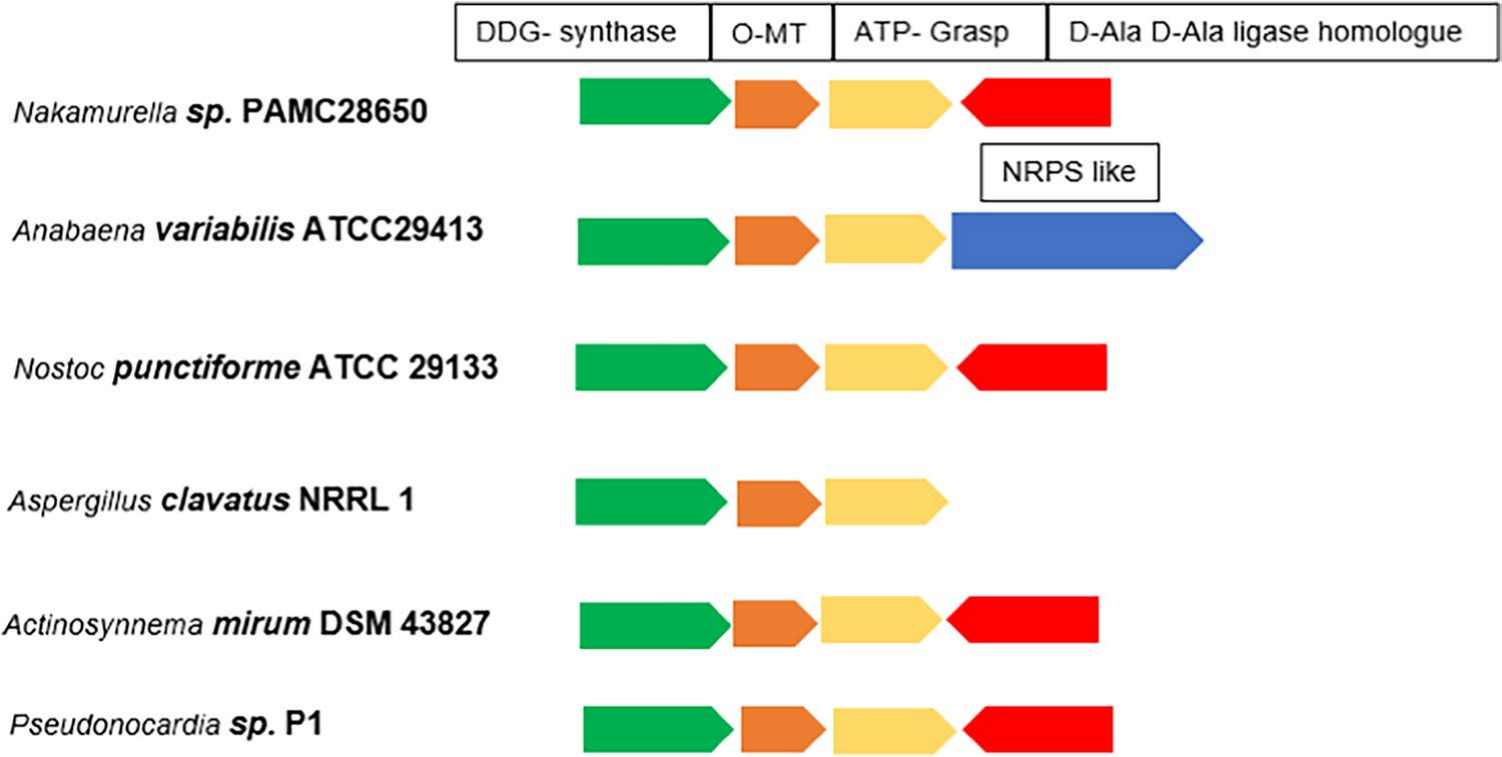
Putative mycosporine-like gene cluster of PAMC28650, compared with other well-characterized bacterial species gene clusters

Mycosporine and MAAs are the small pigment, water-soluble molecules that are generally present in high volume of sunlight. The nucleus of mycosporine-like amino acids is 5-dihydroxy-5-hydroxymethyl cyclohex-1,2-ene ring, with a methoxy group at C-2. Various microorganisms, cyanobacteria, and eukaryotes (fungi, yeast, and microalgae) synthesize them. Some bacteria and actinomycetes are also known to produce mycosporine. *Actinosynnema mirum* DSM43827 and *Pseudonocardia* sp. strain P1, a first report on Gram-positive bacteria to produce mycosporine, show lesser evidence of mycosporine and MAA biosynthesis in bacteria (Miyamoto et al. 2014). As shown by the RAST annotation server, *Nakamurella* sp. PAMC28650 was found to contain gene cluster for MAA biosynthesis. However, the other two *Nakamurella multipartita* DSM44233 and *Nakamurella antarctica* s14-144 lacked the genes. We hypothesize the absence of the gene cluster in the other two species resemble its isolated site, where only *Nakamurella* sp. PAMC28650 was isolated from extreme environment, referring to extreme cold and UV radiation. The enzymes and gene cluster are shown in Fig. 8, where *Nakamurella* sp. PAMC28650 shows exact similarity with the gene cluster of a cyanobacteria *Nostoc punctiforme* ATCC29133 and actinomycetes like *Actinosynnema mirum* DSM43827 and *Pseudonocardia* sp. P1.

Overall, the secondary metabolite gene clusters in *Nakamurella* sp. PAMC28650 signifies its environmental adaptation strategies, but they also carry biotechnological importance. Isorenieratene may ease age-related macular degeneration (AMD) (Chen et al. 2019). Likewise, 3,3′-dihydroxyisorenieratene and isorenieratene are known to help prevent DNA damage in human cells (Martin et al. 2009). MAA structure is composed of cyclohexanone core having two amino acids present in the C1 and C4 positions. In C1 position, amino group is always present while in C4 position, it is substituted by an oxo or an imino moiety that determine the absorption of UV rays. It can dissolve the energy with production of heat without generating ROS (Singh et al. 2021). On the other hand, MAAs are not only known to prevent UV radiation, but also serve as antioxidant, salt stress tolerance, and nitrogen reservoir (Oren and Gunde-Cimerman 2007). Due to photoprotective activity, mycosporine and MAAs are widely used in cosmetics and skin care purposes.

### DNA repair systems in *Nakamurella* species

Based on the isolation environment of *Nakamurella* sp. PAMC28650, our study was directed towards understanding the DNA repair mechanisms. UV radiation can easily damage the DNA in a variety of organisms, including humans. However, bacterial cells may develop different types of DNA repair systems to prevent the UV-induced lesion. Table 6 presents the pathway related to UV-damaged DNA repair systems in *Nakamurella* sp. PAMC28650.

**Table 6:** DNA repair systems along with its genes found in *Nakamurella* sp. PAMC28650

KEGG orthology ID	Repair system	Genes	Protein accession number (CDD)/UniProtKB	Functions
Base excision repair (BER)				
K10563	Bifunctional glycosylases	Fpg	A8A697	Formamidopyrimidine-DNA glycosylase
K05522		Nei	B1X6P5.1	Endonuclease VIII
K10773		NTH	P78549.2	Endonuclease III
K01142		Xth	O26314.1	Exodeoxyribonuclease III
K01151		Nfo	B1X857.1	Endonuclease IV
K02335		Dpol	F416M1.1	DNA polymerase
K01982		Lig	B1IX60.1	RNA ligase family protein
K01247	Monofunctional glycosylases	AlkA	P37878.1	DNA-3-methyladenine glycosylase
K03649		Mug	A1AFY9.1	Specific DNA glycosylase
K01246		Tag	P05100.1	Temporal alpha-galactosidase
K03648		UNG	A3M500.2	Uracil-DNA glycosylase
K03575		MUTY	Q9UIF7.1	Adenine-DNA glycosylase
K03652		MPG	P29372.3	N-Methylpurine DNA glycosylase
Nucleotide excision repair				
K03701		UVRA	P63381.1	Excision nuclease subunit A
K03702		UVRB	A1A921.1	Excision nuclease subunit B
K03723		MFD	P30958.2	Transcription-repair coupling factor
K03703		UVRC	A7ZN37.1	Excision nuclease subunit C
K02335		Dpol	F416M1.1	DNA helicase II
K01972		Lig	B1IX60.1	DNA polymerase
K03657		UVRD	032,215.1	DNA ligase family protein
Homologous recombinant				
K03111		SSB	P0AGE0.2	ssDNA-binding protein
K03553		RecA	B0B8M5.1	DNA recombination/repair protein RecA
K03582		RecB	P08394.1	Exodeoxyribonuclease V subunit RecB
K03583		RecC	P07648.1	Exodeoxyribonuclease V subunit RecC
K03581		RecD	P04993.2	Exodeoxyribonuclease V subunit RecD
K03584		RecO	A1AE95.1	DNA repair protein RecO
K06187		RecR	A1A8D8.1	DNA repair protein RecR
K03629		RecF	Q9RVE0.1	DNA repair protein RecF
K02335		Dpol	P00582.1	DNA polymerase
K02337		Dpolll	B8GWS6.1	Dipeptidyl peptidase 3
K03550		ruvA	A1AC22.1	Holliday junction branch migration complex subunit RubA
K03551		RuvB	A1AC21.1	Holliday junction branch migration complex subunit RubB
Mismatch repair system				
K03657		UVRD	O32215.1	DNA helicase II
K03601		ExoVII	B1XAY4.1	Exodeoxyribonuclease VII large subunit
K03111		PriA	P0AGE0.2	Primosome factor N′
K03111		SSB	P0AGE0.2	ssDNA-binding protein
K02337		Dpollll	B8GWS6.1	Dipeptidyl aminopeptidase III
		Lig	B1X980.1	DNA ligase
Non-homologous end joining				
K10979		Ku	O34859.1	DNA end-binding protein Ku
K1971		Lig	O34398.1	Bifunctional non-homologous end joining LigD

Several studies have reported multiple kinds of DNA repair systems to exist in bacteria. Excision repair systems, including base excision repair (BER), nucleotide excision repair (NER), and mismatch repair (MMR), are the major repair systems in various microorganisms that function by producing glycosylases and polymerases. Other mechanisms include recombination repair, mutagenic repair, and apoptosis. The photolyase-mediated DNA repair system is responsible for the protection of bacterial cells released during photoactivation (Yi and He 2013). We found genes for BER, NER, mismatch repair, and homologous recombination in *Nakamurella* sp. PAMC28650 based on KEGG analysis.

BER system helps protect the bacterial cells from endogenous DNA damage. DNA damage may be caused by hydrolysis or reactive oxygen species (ROS), or UV radiation or strong alkylating agents (Seeberg et al. 1995). Glycosylases are the key enzyme that helps to remove the degraded bases and endonucleases by binding the apurinic/apyrimidinic site (AP site) and breaking the glycosidic bond at DNA 5′ to the abasic site, and DNA polymerases repair it (Dalhus et al. 2009).

NER removes the DNA-distorting lesion ranging from small lesion to large. In bacteria, NER systems involve three different genes (UvrABC present in *E. coli*), which help to recognize the damage, cleave the target site, and carry out the completion of the repair system. The genes like UvrA and UvrB are the energy-independent factors that recognize the lesion, and UvrC and UvrD play the role of the removal of damaged DNA, showing nuclease and helicase activity, respectively. *Nakamurella* sp. PAMC28650 has all the homologous genes related to UvrABCD repair systems and may play a role in the UV-resistant mechanism (Crowley et al. 2006).

The MMR system is activated when the DNA damage is caused by mutation due to error in replication. MutL, MutS (mismatch recognition), and MutH (incision of mismatch strands) genes are mainly responsible for the repair of distorted DNA. However, these three genes are absent in our PAMC28650 strain; however, UVRD helicase II, Exo VII exonucleases, SSB, and DpoIII that play a role in DNA resynthesis, and finally Lig for the ligation of DNA strand, are present.

Homologous recombination is a common pathway for the maintenance and repair of DNA. RecFOR and RecBC are the major pathways for the homologous recombination. *Acinetobacter baumannii* RecA protein played a role in SOS mutagenesis and protection from UV radiation, as well as other stresses, such as oxidative agents and different types of antibiotics (Aranda et al. 2011). *Nakamurella* sp. PAMC28650 consists of all the genes related to this repair mechanism.

## Conclusions

This study presents the complete genome analysis of *Nakamurella* sp. PAMC28650 isolated from the glacier of Mt. Rwenzori, Uganda. We isolated the bacteria under laboratory condition and confirmed that the isolated was *Nakamurella* species depending on the 16S rRNA sequences. The genome analysis showed that the strain possessed several genes related to carbohydrate metabolism, UV resistance, and osmoregulation, which might play an important role in stress management in an extreme environment like glacier. A complete genome analysis suggests that PAMC28650 has a glycogen and cellulose metabolism pathway associated as CAZyme genes. Additionally, isoreineratene (carotenoid) and mycosporine-like amino acid (MAA), the major secondary metabolites that help in the protection against UV radiation, were found in *Nakamurella* sp. PAMC28650. Along with it, the strain also holds the genes responsible for osmotic stress regulation, which is one of the major stress conditions in a cold environment. Overall, we expect that the information provided in this study provides a research scope for understanding of the bacterial ability to withstand extreme cold environment, UV-resistant mechanisms, and osmotic stress with respect to polysaccharide metabolism, secondary metabolite biosynthesis, and osmoregulation. In addition, the highlighted features and genes also show the biotechnological importance of the strain *Nakamurella* sp. PAMC28650.

## Supplementary Information

Below is the link to the electronic supplementary material.

Supplementary file1 (XLSX 15 KB)

Supplementary file2 (DOCX 47 KB)

## Data Availability

The complete genome sequence data that support the findings of this study are openly available in GenBank of NCBI at https://www.ncbi.nlm.nih.gov/, and the deposited GenBank accession number is CP060298.1.
