# Supplementary material for: Complete genome of Nakamurella sp. PAMC28650: genomic insights into its environmental adaptation and biotechnological potential
Source: Funct Integr Genomics. 2022 Dec 23;23(1):18. doi: 10.1007/s10142-022-00937-6 (PMC9789016; doi:10.1007/s10142-022-00937-6)
Supplement: Supplementary file 2 — Supplementary file2 (DOCX 47 KB) [file 10142_2022_937_MOESM2_ESM.docx]

Table S2: The glycogen and cellulose metabolic pathways present in *Nakamurella* sp. PAMC28650.

| **Gene name** | **KEGG orthology ID** | **Enzyme commission number (E.C.)** | **Function** |
| --- | --- | --- | --- |
| CELB | K19357 | 3.2.1.4 | cellulase |
| bcsA | K00694 | [2.4.1.12](https://www.kegg.jp/entry/2.4.1.12) | cellulose synthase |
| bglX | K05349 | [3.2.1.21](https://www.kegg.jp/entry/3.2.1.21) | beta-glucosidase |
| galF | K00963 | [2.7.7.9](https://www.kegg.jp/entry/2.7.7.9) | UTP--glucose-1-phosphate uridylyltransferase |
| glgC | K00975 | [2.7.7.27](https://www.kegg.jp/entry/2.7.7.27) | glucose-1-phosphate adenylyltransferase |
| glgP | K00688 | [2.4.1.1](https://www.kegg.jp/entry/2.4.1.1) | glycogen phosphorylase |
| glgM | K16148 | [2.4.1.342](https://www.kegg.jp/entry/2.4.1.342) | alpha-maltose-1-phosphate synthase |
| glgE | K16147 | [2.4.99.16](https://www.kegg.jp/entry/2.4.99.16) | starch synthase (maltosyl-transferring) |
| glgB | K00700 | [2.4.1.18](https://www.kegg.jp/entry/2.4.1.18) | 1,4-alpha-glucan branching enzyme |
| treX | K01214 | [3.2.1.68](https://www.kegg.jp/entry/3.2.1.68) | isoamylase |
| glgY | K06044 | [5.4.99.15](https://www.kegg.jp/entry/5.4.99.15) | (1->4)-alpha-D-glucan 1-alpha-D-glucosylmutase |
| glgZ | K01236 | [3.2.1.141](https://www.kegg.jp/entry/3.2.1.141) | maltooligosyltrehalose trehalohydrolase |
| malS | K01176 | [3.2.1.1](https://www.kegg.jp/entry/3.2.1.1) | alpha-amylase |
| MGAM | K12047 | [3.2.1.3](https://www.kegg.jp/entry/3.2.1.3) | maltase-glucoamylase |
| SI | K01203 | [3.2.1.10](https://www.kegg.jp/entry/3.2.1.10) | sucrase-isomaltase |
| malZ | K01187 | [3.2.1.20](https://www.kegg.jp/entry/3.2.1.20) | alpha-glucosidase |
| pep2 | K16146 | [2.7.1.175](https://www.kegg.jp/entry/2.7.1.175) | maltokinase |
| treS | K05343 | [5.4.99.16](https://www.kegg.jp/entry/5.4.99.16) | maltose alpha-D-glucosyltransferase |

Table S3: List of CAZymes in three different *Nakamurella* sp. with their putative functions.

| **Gene name** | **KEGG orthology ID** | **Enzyme commission number (E.C.)** | **Function** |
| --- | --- | --- | --- |
| CELB | K19357 | 3.2.1.4 | cellulase |
| bcsA | K00694 | [2.4.1.12](https://www.kegg.jp/entry/2.4.1.12) | cellulose synthase |
| bglX | K05349 | [3.2.1.21](https://www.kegg.jp/entry/3.2.1.21) | beta-glucosidase |
| galF | K00963 | [2.7.7.9](https://www.kegg.jp/entry/2.7.7.9) | UTP--glucose-1-phosphate uridylyltransferase |
| glgC | K00975 | [2.7.7.27](https://www.kegg.jp/entry/2.7.7.27) | glucose-1-phosphate adenylyltransferase |
| glgP | K00688 | [2.4.1.1](https://www.kegg.jp/entry/2.4.1.1) | glycogen phosphorylase |
| glgM | K16148 | [2.4.1.342](https://www.kegg.jp/entry/2.4.1.342) | alpha-maltose-1-phosphate synthase |
| glgE | K16147 | [2.4.99.16](https://www.kegg.jp/entry/2.4.99.16) | starch synthase (maltosyl-transferring) |
| glgB | K00700 | [2.4.1.18](https://www.kegg.jp/entry/2.4.1.18) | 1,4-alpha-glucan branching enzyme |
| treX | K01214 | [3.2.1.68](https://www.kegg.jp/entry/3.2.1.68) | isoamylase |
| glgY | K06044 | [5.4.99.15](https://www.kegg.jp/entry/5.4.99.15) | (1->4)-alpha-D-glucan 1-alpha-D-glucosylmutase |
| glgZ | K01236 | [3.2.1.141](https://www.kegg.jp/entry/3.2.1.141) | maltooligosyltrehalose trehalohydrolase |
| malS | K01176 | [3.2.1.1](https://www.kegg.jp/entry/3.2.1.1) | alpha-amylase |
| MGAM | K12047 | [3.2.1.3](https://www.kegg.jp/entry/3.2.1.3) | maltase-glucoamylase |
| SI |  | [3.2.1.10](https://www.kegg.jp/entry/3.2.1.10) | sucrase-isomaltase |
| malZ | K01187 | [3.2.1.20](https://www.kegg.jp/entry/3.2.1.20) | alpha-glucosidase |
| pep2 | K16146 | [2.7.1.175](https://www.kegg.jp/entry/2.7.1.175) | maltokinase |
| treS | K05343 | [5.4.99.16](https://www.kegg.jp/entry/5.4.99.16) | maltose alpha-D-glucosyltransferase |

Table S4: Average nucleotide identity (ANI) analysis performed between the species having complete genomes within *Frankineae* suborder.

|  | *Nakamurella* sp. s14-144 | *Nakamurella*  *Multipartita* DSM 44233 | *Epidermidibacterium keratini* EP1-7 | *Acidothermus cellulolyticus* 11B | *Frankia inefficax* strain Eul1c | *Frankia sp*. EAN1pec | *Frakia casuarinae* strain Ccl3 | *Frankia alni* str. ACN14A | *Candidatus Frankia datiscae* strain Dg1 |
| --- | --- | --- | --- | --- | --- | --- | --- | --- | --- |
| *Nakamurella* sp. PAMC28650 | 69.81 | 73.34 | 68.34 | 67.20 | 68.71 | 68.51 | 68.65 | 68.69 | 68.49 |
| *Nakamurella* sp. s14-144 | - | 69.91 | 66.62 | 65.98 | 66.81 | 66.64 | 66.45 | 66.69 | 66.44 |
| *Nakamurella*  *Multipartita* DSM 44233 | - | - | 68.72 | 67.69 | 69.47 | 69.00 | 68.95 | 69.29 | 69.47 |
| *Epidermidibacterium keratini* EP1-7 | - | - | - | 67.54 | 68.68 | 68.71 | 68.41 | 68.68 | 68.45 |
| *Acidothermus cellulolyticus* 11B | - | - | - | - | 68.73 | 68.64 | 68.59 | 68.82 | 68.93 |
| *Frankia inefficax* strain Eul1c | - | - | - | - | - | 75.66 | 75.05 | 75.27 | 74.63 |
| *Frankia* sp. EAN1pec | - | - | - | - | - | - | 77.24 | 76.78 | 75.73 |
| *Frakia casuarinae* strain Ccl3 | - | - | - | - | - | - | - | 81.04 | 75.79 |
| *Frankia alni* str. ACN14A | - | - | - | - | - | - | - | - | 75.78 |
| *Candidatus Frankia datiscae* strain Dg1 | - | - | - | - | - | - | - | - | - |

Table S5: Cold shock protein encoding genes and mycosporine synthetic gene clusters present in *Nakamurella* sp. PAMC28650

| **Cold shock protein** | RAST ID | CSP genes present in *Nakamurella* sp. PAMC28650 | Protein ID/UniprotKB | Reported function | Highest similarity strain | Amino acid similarity |
| --- | --- | --- | --- | --- | --- | --- |
|  | Peg.1377 | cspA | P54581.1 | Major cold shock protein | *Arthrobacter globiformis* | 73.13% |
|  | Peg.3911 | cspA | A0R5E1.1 | Major cold shock protein | *Mycolicibacterium smegmatis* Mc2 155 | 70.15% |
|  | Peg.5207 | CspE | P62169.1 | Regulation and expression of stress response protein | *Bacillus anthracis* | 57.14% |
| **Mycosporine biosynthesis**  **Genes** | [peg.](https://rast.nmpdr.org/seedviewer.cgi?page=BrowseGenome&feature=fig\|2762325.4.peg.4126)4122 | MysD | A5FGN3.1 | D-alanine—D-alanine ligase | *Flavobactrtium johnsoniae* UW101 | 30.17% |
|  | [peg.](https://rast.nmpdr.org/seedviewer.cgi?page=BrowseGenome&feature=fig\|2762325.4.peg.4126)4123 | MysC | - | ATP-grasp ligase forming mycosporine-glycine | - | - |
|  | [peg.](https://rast.nmpdr.org/seedviewer.cgi?page=BrowseGenome&feature=fig\|2762325.4.peg.4126)4125 | MysB | Q86IC9.1 | O-methyltransferase | *Streptomyces mycarofaciens* | 40.80% |
|  | [peg.](https://rast.nmpdr.org/seedviewer.cgi?page=BrowseGenome&feature=fig\|2762325.4.peg.4126)4126 | MysA | Q3M6C3.1 | Demethyl 4-deoxygadusol synthase | *Trichormus variabilies* ATCC 29413 | 62.47% |
| ^RAST annotated amino acid sequences were analyzed using UniprotKB database of NCBI. (-) sign indicates information is not available in the database.^ | | | | | | |

Table S6: Isorenieratene related genes and its putative function.

| Locus tag | Protein ID | Gene | Location | Putative function |
| --- | --- | --- | --- | --- |
| SGR_6824 | BAG23653.1 | crtY | 8,206,573 - 8,207,814 | Lycopene cyclase |
| SGR_6825 | BAG23654.1 | crtT | 8,207,811 - 8,208,539 | Methyl transferase |
| SGR_6826 | BAG23655.1 | crtU | 8,208,536 - 8,210,092 | B-carotene desaturase/methylase |
| SGR_6827 | BAG23656.1 | crtV | 8,210,128 - 8,211,144 | Methyl esterase |
| SGR_6828 | BAG23657.1 | crtB | 8,211,141 - 8,212,169 | Phytoene synthase |
| SGR_6829 | BAG23658.1 | crtI | 8,212,166 - 8,213,689 | Phytoene dehydrogenase |
| SGR_6830 | BAG23659.1 | crtE | 8,213,686 - 8,214,963 | Geranylgeranyl pyrophosphate synthase |
| ^All the information were taken from the antiSMASH database.^ | | | | |


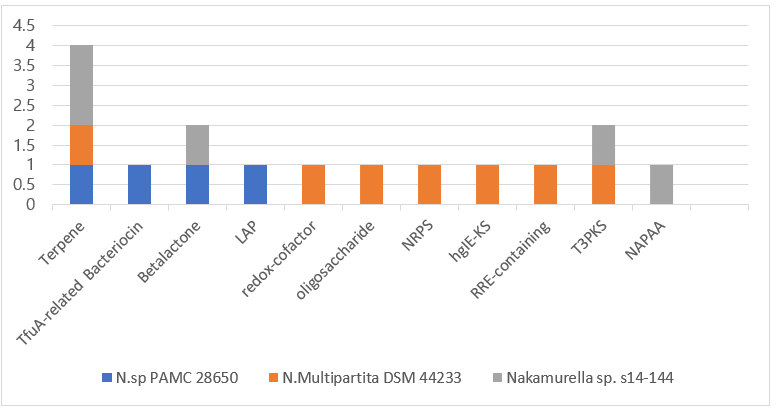


Supplementary Fig. S1. Secondary metabolites gene clusters of three different *Nakamurella* sp. separated by different color.
